# Effects of a Combined Nutritional and Physical Training Program Approach in a Case of Facioscapulohumeral Dystrophy: A One-Year Follow-Up

**DOI:** 10.1155/carm/7873892

**Published:** 2025-07-31

**Authors:** Venere Quintiero, Oscar Crisafulli, Jessica Lacetera, Giorgio Bottoni, Massimo Negro, Rossella Tupler, Emanuela Lavaselli, Giuseppe D'Antona

**Affiliations:** ^1^CRIAMS-Sport Medicine Centre Voghera, University of Pavia, Voghera 27058, Italy; ^2^Department of Life Sciences, University of Modena and Reggio Emilia, Modena 41125, Italy; ^3^Department of Public Health, Experimental and Forensic Medicine, University of Pavia, Pavia 27100, Italy

**Keywords:** body composition, FSHD, leucine, nutrition, physical exercise, proteins, resting metabolic rate

## Abstract

**Background/Objectives:** Facioscapulohumeral dystrophy (FSHD) patients experience a progressive loss of fat free mass (FFM) and increase of fat mass (FM). Such an occurrence may lead to an impaired physical efficiency. A personalized diet, combined with a physical exercise program, may improve body composition, and potentially reduce functional limitations. Here, we present the case of a nineteen-years-old male clinically and genetically characterized FSHD patient who underwent a one-year nutritional and training intervention aimed at contrasting the disease-induced body composition modifications and associated negative sequelae.

**Methods:** Baseline assessments included dietary intake (nutritional anamnesis), body composition (bioimpedance analysis), biochemical parameters (blood tests), resting metabolic rate (RMR; measured by indirect calorimetry), physical efficiency, and quality of life (Checklist Individual Strength Fatigue and Functional Assessment Chronic Illness Therapy Fatigue). Based on the initial findings (insufficient daily caloric intake, inadequate leucine distribution, and nonphysiological glycemia), a personalized nutritional (50% carbohydrates, 30% fats and proteins at 1.5 g/kg of body weight/day, with leucine intake of 1–3 g per meal) and supplementation (11 g/day of essential amino acids) plan was prescribed, alongside a physical training program composed by two resistance and one aerobic exercise sessions per week.

**Results:** After one year, improvements in body composition (FFM +6.9 kg, body cell mass +3.3 kg, FM −2.1 kg), RMR (+309 kcal/day), fasting glycemia (−1.6 mmol/L), perceived physical efficiency (diminished perceived fatigue), and quality of life were reported.

**Conclusions:** Our results suggest that a tailored dietary intervention, when combined with an appropriate training program, could represent a promising long-term strategy for contrasting disease-related physical deconditioning in FSHD. These findings encourage further research on this approach in a larger cohort of patients.

## 1. Introduction

Facioscapulohumeral muscular dystrophy (FSHD) is one of the most common hereditary muscular dystrophies, with an estimated prevalence of 1–9/100000 [1]. Although the clinical presentation is highly variable [2], it is generally characterized by progressive loss of fat free mass (FFM), accompanied by an increase in FM [3]. Weakness of the facial, shoulder, and upper arm muscles represents the primary clinical manifestations, but, in some cases, the trunk and lower limbs may also be affected [4–7]. Overtime, these symptoms may possibly cause fatigue, reduced mobility, functional impairments, and a diminished quality of life [7–9]. Interestingly, lifestyle-related approaches, including nutritional interventions and physical exercise (either alone or combined), have been indicated as effective strategies for contrasting physical deconditioning in various muscular diseases [10–14]. For instance, despite the limits of specificity due to the wide variability in clinical manifestations [15, 16], nutritional recommendations have been proposed for spinal muscular atrophy (SMA) and Duchenne muscular dystrophy (DMD) [17, 18], while no specific dietary guidelines exist for FSHD. However, given the peculiar changes in body composition [3], it is plausible that a diet providing an adequate amount of high biological value proteins and of leucine at each meal, combined with a physical training program, could promote and enhance muscle protein synthesis (MPS) [19, 20] and help preserve muscle function [21, 22]. Regarding physical exercise, previous studies have investigated the effects of aerobic [23, 24] and resistance training [25, 26] on patients with FSHD, reporting some ameliorations, in terms of aerobic fitness [23, 24], muscle endurance [27], strength [25], and quality of life [26]. However, these studies focused on the effects of physical exercise alone, while the combined use of a personalized nutritional program and exercise has yet to be tested in these patients. Importantly, beyond its potential usefulness in improving body composition and physical efficiency, it could also reduce the onset of risk factors associated with the aforementioned alterations in body composition, such as hyperglycemia, and insulin resistance (IR) [28], as already observed in other populations [29]. In the present study, we tested a combined nutritional and aerobic/anaerobic training program in a nineteen-year-old male affected by FSHD, evaluating its effects on body composition, metabolic parameters, physical efficiency, and quality of life.

## 2. Case Presentation

**Figure 1:**
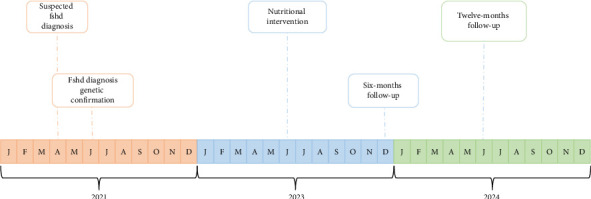
Timeline of patient's salient events. Letters refer to the initials of each month (“J” stands for January, “F” stands for February, “M” stands for March and so on).

The patient was a nineteen-year-old male diagnosed with FSHD. In 2021, at the age of 18, following the onset of typical symptoms, such as fatigue and weakness in the facial and upper limb muscles, he underwent genetic and molecular investigations highlighting the presence of 4q35 allele of 35 kb, which is associated with FSHD [30]. Subsequent clinical evaluations confirmed the diagnosis, and the patient has been allocated to subcategory A3, since presenting weakness of the upper facial muscles in combination with impaired upper limb abduction and winged scapula [31]. In June 2023, the patient sought care at our Sport Medicine Center, searching for an effective way to ameliorate his physical condition, referred to as progressively damaged by the disease. He was subjected to a program composed of a dietary plan, tailored to his caloric and nutrient requirements, and a proper physical training managed by a qualified operator of our Center (GB) (Figure 1).

### 2.1. Patient Evaluations

The patient provided written informed consent to participate in the study, which was approved by the Lombardy Territorial Ethics Committee 6, under protocol number 0006176/24. At baseline (T_0_) the patient underwent dietary intake evaluation, body composition analysis, biochemical, and resting metabolic rate (RMR) assessments. Additionally, validated questionnaires were used to evaluate perceived physical efficiency and quality of life. After 6 and 12 months (T_1_ and T_2_, respectively) from baseline, body composition analysis, biochemical parameters, RMR, physical efficiency, and quality of life assessments were repeated, and the results were compared to evaluate the effectiveness of the combined dietary and physical training intervention. The results are summarized in Tables 1 and 2.

#### 2.1.1. Dietary Intake Evaluation

Before the intervention, an exhaustive nutritional anamnesis was conducted by two experienced dietitians (VQ and EL). Data were converted into energy, macronutrients, and micronutrients values using the Food Composition Database of the European Institute of Oncology. Nutritional adequacy was assessed according to the Reference Intake Levels of Nutrients and Energy (LARN) [32], excluding protein intake (as described in Section 2.2).

#### 2.1.2. Anthropometry and Bioelectrical Impedance Analysis

Body weight (BW, kg) and height (H, m) were obtained, and body mass index (BMI) was calculated using the formula BMI = BW (kg)/H^2^ (m^2^). Bioelectrical impedance analysis (BIA 101, Akern, Florence, Italy) was performed to investigate body composition. After cleaning the patient's skin with an isotropic alcohol, two pairs of electrodes were placed on the back of the hand and foot of the same side, asking the patient to keep the arms and legs away from the trunk. FM, FFM, and its metabolically active subcomponent, the body cell mass (BCM), parameters were considered. To estimate FFM, the Bodygram PRO v.3.0 software applied the equation validated by Sun et al. [33]. FM was calculated by subtracting FFM values from BW, while BCM was determined using the formula validated by Kotler et al. [34, 35].

#### 2.1.3. Blood Analysis

Blood analysis was conducted before each evaluation. Notably, among the biochemical parameters, only blood glucose was found to be above normal level [36]. Given that hyperglycemia and IR are common consequences of the peculiar above-mentioned alterations of body composition (increased FM and reduced FFM) [28], IR was, therefore, investigated using the homeostasis model assessment (HOMA) equation, as validated by Matthews et al. [37]:

(1) $$\begin{array}{c} \text{HOMA}-\text{IR}=\text{fasting glucose }\left(\text{mmol}/L\right)\times \frac{\text{fasting insulin }\left(\mu \text{UI}/\text{mL}\right)}{22.5}. \end{array}$$

#### 2.1.4. RMR

RMR was measured using indirect calorimetry technique (Quark PFT, Cosmed, Italy) at 08:00 a.m., following an overnight fast and in a thermoneutral environment (23°C). The subject was instructed to remain relaxed and awake throughout the test. Data collection lasted for 20 min (min), with a 5 min run-in time for the stabilization of fraction of expired VCO_2_, between 1.0% and 1.2%, assessed through a Canopy Hood (Canopy, Cosmed, Italy) [38].

#### 2.1.5. Perceived Physical Efficiency and Quality of Life Questionnaires

Checklist Individual Strength (CIS20R) subjective fatigue subscale and Functional Assessment Chronic Illness Therapy Fatigue (FACIT-F) are self-reported questionnaires used to evaluate physical efficiency and quality of life in several cohorts of patients [39, 40]. The subjective fatigue subscale of the CIS20R was employed to assess patient's perceived fatigue; the total score ranged from 8 to 56, with a score ≥ 35 indicating sever fatigue [41]. FACIT-F, instead, was used to investigate patient's quality of life across several domains: physical well-being (PWB; total score 0–28, with negatively worded items so that lower scores reflect greater PWB), social/family well-being (SWB; total score 0–28, with higher scores indicating greater SWB), emotional well-being (EWB; total score 0–24, with negatively worded items, so that lower scores reflect greater EWB), functional well-being (FWB; total score 0–28, with higher scores indicating greater FWB), and fatigue (F; total score 0–52, with higher scores indicating better quality of life) [42].

### 2.2. Dietary Intervention

Baseline nutritional anamnesis revealed a daily caloric intake lower than the required needs (with a mean of 2151 kcal/day), and an inadequate distribution of leucine across meals (mainly consumed at lunch and dinner). However, daily requirements for vitamins and minerals were met. The other nutrient contributions were as follows: 51.2% carbohydrates, 8.4% sugars, 27.7% fats, 7.4% saturated fats, 1.5 g of proteins/kg of BW/day (21.1% of total energy). Additionally, the fiber intake was 26 g/day, and the mean cholesterol intake was 261.1 mg/day (Table 2). Considering a RMR of 1.762 kcal/day, a personalized isocaloric diet of 2600 kcal/day was developed, based on the estimated daily caloric, macronutrient (excluding proteins), and micronutrient requirements by age and gender [32]. For the assessment of protein needs, the nutritional recommendations for the management of sarcopenia [43, 44] were taken into account. Although specific data are still lacking, given the protein catabolism and progressive FFM loss, peculiar of FSHD [4, 9, 45], we have hypothesized that, similar to sarcopenic individuals, the patient may benefit from a higher protein intake (set at 1.5 g/kg of BW/day) compared to the healthy population [32]. Mean protein quantity usually consumed by the patient before the intervention was comparable to the specific requirement we hypothesized above. However, daily leucine distribution across the various meals was found to be inadequate. Leucine is an essential amino acid (EAA) and a key regulator of muscle protein synthesis via activation of the mammalian target of rapamycin kinase complex 1 (mTORC1) [46]. Ensuring an adequate supply of this EAA (1–3 g) in each meal is essential for optimizing muscle protein anabolism [19, 20], especially in hypercatabolic states [47], such as FSHD. Although the total leucine intake remained approximately the same compared to T_0_, its distribution across meals was improved. In addition to the dietary adjustments, the patient began a nutritional supplementation regimen to further support MPS (Aminotrofic-NE, Errekappa, 11 g/day of EAAs). Adherence to the nutritional plan was monitored using a 3-day weighed food diary (including two weekdays and one weekend day) and the 24-h recall method [48]. A summary of the nutritional intervention is presented in Table 2.

### 2.3. Training Program

The training program consisted of two weekly sessions of resistance exercises, each lasting 60 min, and one weekly session of aerobic exercises lasting 45 min. The resistance training targeted the core and abdominal muscles (hand-foot plank and side plank [2 sets for 30 s each]), lower limbs [squat (4 sets of 10 reps each), hip thrust (3 sets of 10 reps each), calf standing (3 sets of 12 reps each), and upper limbs, including the shoulders (lateral rises, 3 sets of 15 reps each), biceps (dumbbell curl, 3 sets of 10 reps each), and triceps (dumbbell French press, 3 sets of 10 reps each). Recovery time between exercises was 1 min and 30 s. The aerobic training was performed on a bike, maintaining a heart rate ranging from 40% to 50% of theoretical maximal heart rate, estimated with the age-based prediction equation (220 beats per minute—age), validated by Fox et al. [49]. The exercises program lasted 1 year, a period widely sufficient to induce adaptations to both anaerobic [50] and aerobic training [51].

## 3. Discussion

In author's knowledge, this is the first study analyzing the effectiveness of a nutritional and supplementation plan, combined to a physical training program, on the physical condition of a patient with FSHD. Despite starting from a favorable condition (except for fasting glucose), our results showed improvements in body composition, some plasma parameters, RMR, physical efficiency and quality of life, particularly at T_2_. For instance, after 1 year, body composition analysis revealed an increase in FFM and BCM, coherently with the augmentation of RMR [52]. Such results, although not generalizable, seem coherent with our original hypothesis for which a FSHD patient might benefit from a higher intake of high biological value proteins, compared to the healthy population [32], and from an optimal distribution of leucine across the various meals of the day [19, 20]. It should be noted that the mean daily leucine intake remained quite similar between T_0_ and T_1_, but its distribution throughout the day changed to ensure that each meal provided 1–3 g of leucine, an amount considered optimal for stimulating MPS [19, 20]. In fact, leucine intake exceeding this range, as in the case of our patient prior to the intervention, does not provide additional benefits for MPS [53].

In addition to the nutritional plan, our intervention included an aerobic/anaerobic training program. Aerobic training is highly recommended for weight and FM loss [54], but it also provides benefits in enhancing physical efficiency in both general [55] and FSHD populations [23, 24, 27]. In contrast, resistance exercises are primarily associated with muscle hypertrophy [50, 56]. Our results seem to be coherent with these observations, since beside the augmentation in FFM and BCM, a reduction in FM was also observed. Notably, at T_1_, FM increased, possibly due to the initial adaptation to the new nutritional plan [57], consisting of an isocaloric diet (averaging of 2600 kcal/day) relative to the patient's daily needs, but hypercaloric (at least initially) compared to his usual diet (mean of 2151 kcal/day). This initial caloric surplus could have induced an adaptation phase, as evidenced by increases in BW, FFM, BCM, and FM (+3.8 kg, +2.5, +0.6 and + 1.3 from baseline, respectively). As BW, FFM, and BCM gradually increased, RMR and daily energy expenditure (DEE) also potentially increased [52, 57], until a new equilibrium was reached, where DEE equaled daily energy intake [57]. Between T_1_ and T_2_, in fact, BW stabilized, and FM decreased, as previously explained.

At baseline, blood glucose (6.2 mmol/L) and HOMA-IR (2.8) indicated early signs of IR [36, 37]; however, the underlying factors contributing to this tendency are unclear. Although it is known that FM accumulation and energy imbalance are common contributors to IR [58], the patient's baseline results would not indicate such factors as determinants (see Tables 1 and 2, respectively). Hence, other uninvestigated factors may be responsible for such values. For instance, inflammation, common trait of FSHD [59], is also a well-recognized determinant of IR [58] and may have played a role in this case. However, since no inflammatory markers were evaluated, this hypothesis remains purely suggestive. In addition, and/or in alternative to the role of IR, elevated blood glucose levels may also be due to enzymatic dysregulation. In fact, a recent study by Moriggi and colleagues [60] revealed several alterations in glycolytic enzymes in both mild and severe FSHD patients, resulting in impaired glycolysis. Such an impairment may lead to glucose accumulation and contribute to hyperglycemia. However, once again, due to the lack of specific data, such hypothesis remains speculative at this stage. Nevertheless, regardless of the cause, our approach improved fasting glucose and insulin levels at both T_1_ and T_2_, along with HOMA-IR (Table 1), consistent with previous observations that exercise, and nutrition are key factors in managing IR [29, 61, 62].

The initial results of the CIS20R subjective fatigue and FACIT-F subscales (summarized in Table 1) indicated a good baseline condition. The absence of a markedly deconditioned state at T_0_ may have contributed to the patient's compliance and motivation to further improve physical condition to contrast disease progression. In parallel with positive changes in body composition and metabolic parameters, improvements in quality of life and physical efficiency were also observed, in line with previous studies on other cohorts of patients, subjected to nutritional and/or exercise intervention [12, 14, 63, 64]. At T_2_, the PWB and EWB score reductions, and SWB, FWB, and F score increases indicate an overall amelioration of perceived physical condition, coherently with the CIS20R subjective fatigue score indicating a reduction in perceived fatigue.

### 3.1. Limitations

This study suffers from some limitations. First, while promising, our results refer to a single case and cannot be generalizable to other patients with FSHD. Furthermore, given the slow progression of the disease, the one-year follow-up period may be relatively brief. Sustained adherence over a longer period could offer valuable insights into the long-term effects of the combined exercise and nutritional protocols. Moreover, although BIA has been previously used in several studies on dystrophic patients [65–67], including FSHD [9, 45], it relies on population-specific predictive equations, which, when applied to cohorts with different characteristics, could provide biased results [68]. Nonetheless, the observed increase in RMR, unbiased data, seems to be coherent with the observed FFM and BCM augmentation [52], suggesting the substantial reliability of the evaluation. Another limitation is that the level of physical activity, a factor known to influence body composition, biochemical and metabolic parameters [61, 69], was not investigated. Future studies will take advantages from the use of accelerometers as detectors of physical activity energy expenditure [70]. Additionally, the 24-h recall used to assess compliance with the nutritional plan is subject to biases due to memory errors, as it is a retrospective method. In contrast, the 3-day weighed food diary, a prospective method, helps mitigate this limitation, although requiring collaboration and commitment from the respondent [48].

## 4. Conclusions

Our results suggest that a tailored nutritional intervention combined with a physical training program could represent a promising approach for improving physical condition in a FSHD patient, over the long term. These data encourage the implementation of the proposed methodology in a larger patient cohort and suggest the importance of a healthy lifestyle to contrast the disease-induced negative sequelae.

## Figures and Tables

**Table 1:** Summary of anthropometric, body composition, biochemical and metabolic characteristics, and questionnaires scores of the patient at baseline (T_0_) and follow-up assessments (T_1_ and T_2_).

	T_0_	T_1_	T_2_	Δ% (T_0_–T_2_)
Anthropometric variables				
Weight (kg)	75.8	79.6	80.6	+6.3%
Height (m)	1.84	1.84	1.84	—
BMI (kg/m^2^)	22.4	23.5	23.9	+6.7%
Body composition variables				
FFM (kg)	66.9	69.4	73.8	+10.3%
BCM (kg)	41.2	41.8	44.5	+8.0%
FM (kg)	8.9	10.2	6.8	−23.6%
Biochemical and metabolic parameters				
Blood glucose level (mmol/L)	6.2	4.9	4.6	−25.8%
Blood insulin level (μUI/mL)	10.2	6.6	6.1	−40.2%
HOMA-IR index	2.8	1.4	1.2	−57.1%
RMR (kcal/day)	1762	1892	2071	+17.5%
Questionnaires subscales scores				
CIS20R subjective fatigue (score 8–56)	18	17	16	−11.1%
FACIT-F:				
PWB (score 0–28)	3	2	1	−66.7%
SWB (score 0–28)	22	23	27	+22.7%
EWB (score 0–24)	6	8	2	−66.7%
FWB (score 0–28)	24	26	28	+16.7%
F (score 0–52)	46	48	48	+4.3%

*Note:* Δ% (T_0_–T_2_) indicates the difference, expressed in %, between values at baseline and twelve-months follow-up assessment. For CIS20R, a score ≥ 35 indicate severe fatigue; for PWB, and EWB scores, higher scores reflect greater severity; for SWB, FWB and F, higher scores reflect better quality of life. HOMA-IR, homeostasis model assessment for insulin resistance; CIS20R, Checklist Individual Strength; SWB, social/family well-being; F, fatigue; kg, kilograms; m, meter; mmol, millimole; L, liter; μUI, micro-international units; mL, milliliter.

Abbreviations: BCM, body cell mass; BMI, body mass index; BW, body weight; EWB, emotional well-being; FACIT-F, Functional Assessment Chronic Illness Therapy (Fatigue); FFM, fat free mass; FM, fat mass; FWB, functional well-being; PWB, physical well-being; RMR, resting metabolic rate.

**Table 2:** Mean daily nutritional composition, leucine distribution, and supplementation of patient's habitual diet (baseline) and of the nutritional plan (dietary intervention).

	Baseline	Dietary intervention
	Mean ± SD	Mean
*Energy and nutrients contribution*		
Total daily energy (kcal/day)	2151 ± 558	2600
Protein (% of total daily energy)	21.1 ± 11.3	17.5
Protein (g/kg/day)	1.5 ± 0.4	1.5
Leucine (g/day)	10.6 ± 2.4	11.3
Leucine (g/day at breakfast)	0.9 ± 0	1.5
Leucine (g/day at morning snack)	0 ± 0	1.1
Leucine (g/day at lunch)	4.9 ± 2.3	3
Leucine (g/day at afternoon snack)	0 ± 0	2.8
Leucine (g/day at dinner)	4.8 ± 2.4	2.9
Fat (% of total daily energy)	27.7 ± 6.9	31.4
Saturated fat (% of total daily energy)	7.4 ± 3.9	4.8
Carbohydrates (% of total daily energy)	51.2 ± 8.4	51.1
Simple sugars (% of total daily energy)	8.4 ± 1.3	9.3
Fiber (g/day)	26 ± 2.4	30.7
Cholesterol (mg/day)	261.1 ± 51.9	137.5

*Supplementation*		
Aminotrofic-NE (g/day)	—	11

*Note:* Data are expressed as mean and, in the case of pre dietary intervention, as standard deviation (SD). For the leucine meals content of dietary intervention, the supplementation plan was considered. kcal, kilocalories; g, grams; kg, kilograms; mg, milligrams.

## Data Availability

The data that support the findings of this study are available on request from the corresponding author. The data are not publicly available due to privacy or ethical restrictions.
